# Protective effects of fisetin on ovarian ischemia-reperfusion injury in rats via modulation of the TLR4-MyD88-TRAF6 signaling pathway

**DOI:** 10.1590/acb405925

**Published:** 2025-09-19

**Authors:** Qianqian Gao, Dan Zhao, Wencong He

**Affiliations:** 1 China Three Gorges University – The First College of Clinical Medical Science – Yichang – China; 2 Yichang Central People’s Hospital – Department of Obstetrics and Gynecology – Yichang – China.; 3 China Three Gorges University – Institute of Obstetrics and Gynecology – Yichang – China.

**Keywords:** Ovary, Ischemia, Reperfusion Injury, Oxidative Stress, Apoptosis

## Abstract

**Purpose:**

To investigate the protective effects of fisetin on ovarian ischemia-reperfusion injury in rats, focusing on the modulation of the TLR4-MyD88-TRAF6 signaling pathway.

**Methods:**

Wistar rats randomly divided into different groups received oral administration of fisetin (5, 10 and 15 mg/kg). Body weight and ovary weight were measured. Hormones, hematological, antioxidant, cytokines, inflammatory and apoptosis parameters were assessed. Histological and histopathologic evaluations were conducted on ovary tissue. Different mRNA expressions were estimated.

**Results:**

Dose dependent treatment significantly improved body and ovary weight along with alteration in hematological parameters such as red blood cells, white blood cells, hemoglobin, platelet, lymphocyte; antioxidant parameters, including malonaldehyde, superoxide dismutase, glutathione, glutathione peroxidase, catalase; cytokines viz., tumor necrosis factor-α (TNF-α), interleukin-1β (IL-1β), interleukin-6 (IL-6), interleukin-10 (IL-10), interleukin-18 (IL-18); inflammatory parameters such as cyclooxynase-2, inducible nitric oxide synthetase (iNOS), prostaglaindin, nuclear kappa B factor (NF-κB), C reactive protein and apoptosis parameters, including Bcl-2, Bax, and caspase-3. Fisetin treatment significantly (p < 0.001) altered the mRNA expression of TNF-α, IL-6, IL-1β, IL-10, Bax, Bcl-2, caspase-3, toll-like receptor 4, myeloid differentiation primary response protein 88, tumor necrosis factor receptor-associated factor 6, endothelial nitric oxide synthase, iNOS, NF-κB, inhibitor of kappaB kinase alpha, heme oxygenase-1, and nuclear factor erythroid 2-related factor 2. Fisetin treatment altered the hemorrhage, vascular proliferation, polymorphonuclear leukocyte, edema, vascular congestion, and apoptosis.

**Conclusion:**

Fisetin ameliorated the ovary injury against ovarian ischemia-reperfusion via conquering TLR4-MyD88-TRAF6 signaling pathway.

## Introduction

Ovarian torsion is a significant cause of severe morbidity in females, particularly in childhood and adolescence, as it disrupts blood flow to the ovarian tissue^1^,^2^. Blood flow to the ovaries can be restored through surgical detorsion, but this procedure may lead to ischemia-reperfusion (I/R) injury. Ovarian torsion is a medical emergency condition, and timely surgical intervention is crucial to prevent irreversible ischemic damage to the ovary^1^. Timely diagnosis and intervention are essential for preserving ovarian function and maintaining fertility. The main symptom of ovarian torsion, also known as adnexal torsion, is sudden abdominal pain.

Studies indicate that ovarian torsion can occur in post-menopausal, pre-pubertal, and pregnant women. Consequently, the surgical procedure to address this condition may lead to vascular injury of the ovary^3^. Hypoxia induced via reduction blood flow leads to ischemic tissue injury^4^. After detorsion, the excessive production of reactive oxygen species (ROS) and reactive nitrogen species, coupled with the accumulation of activated neutrophils, contributes to reperfusion injury^5^.

Reperfusion of hypoxic tissue, while necessary for restoring blood flow and oxygen, can initiate a new pathological process that may lead to the additional tissue injury^3^. Reperfusion injury exacerbates the initial ischemic damage at the cellular level, especially through the production of ROS. These ROS, produced by polymorphonuclear leukocytes (PMNL) and other cells, can cause tissue damage and lead to both local and systemic complications during I/R injury^6^.

During the I/R injury process, there is a notable increase in ROS such as superoxide radicals, hydrogen peroxide, and hydroxyl radicals in organs like the kidneys, ovaries, brain, testis, and myocardium. These ROS can damage cellular structures, including proteins, membrane lipids, and nucleic acids^3^,^6^. In response to inflammatory signals, ROS and other inflammatory mediators are released from vascular cells, plasma, and leukocytes^3^,^6^. Elevated levels of ROS can cause oxidative damage to cellular components such as lipids, proteins, and deoxyribonucleic acid (DNA), compromising tissue function and viability.

Studies have shown that increased synthesis of malonaldehyde (MDA), a key marker of oxidative stress, significantly contributes to tissue injury. Glutathione (GSH), an antioxidant, protects tissues from ROS-induced damage and helps maintain the balance between antioxidants and oxidants. Malondialdehyde (MDA), a key byproduct of polyunsaturated fatty acid peroxidation with toxic properties, is widely used as a marker for evaluating oxidative stress. Nitric oxide (NO) is synthesized by three isoforms of nitric oxide synthase: endothelial (eNOS), neuronal (nNOS), and inducible (iNOS)7. Previous studies indicate that eNOS and iNOS are the primary enzymes that play a crucial role in the rat ovaries. An excessive production of intracellular NO levels can trigger the toxic events, leading to cell death, particularly through the generation of free radicals and the formation of harmful products like peroxynitrite^8^. Previous studies have shown that various agents with antioxidant properties can mitigate oxidative stress induced by I/R injury in the ovary. Furthermore, the increase in cytokine levels during reperfusion injury suggests that anti-inflammatory agents may be more effective than antioxidants in preventing ischemic damage^7^.

Heme oxygenase-1 (HO-1) is a vital enzyme that mediates the first and rate-limiting step of heme degradation, leading to the formation of biliverdin, carbon monoxide, and free iron^9^. This enzyme provides significant protection in various disease models through its anti-inflammatory effects and regulation of cell proliferation. HO-1 is crucial in modulating immune-mediated inflammatory responses and plays an important role in several ovarian physiological processes, including steroidogenesis. A deficiency in HO-1 has been associated with ovarian damage and disrupted ovulation, underscoring its vital contribution to ovarian function and overall reproductive health^10^,^11^. Refaie and El-Hussieny^8^ have demonstrated the protective effects of HO-1 in various models of I/R injury.

Fisetin is a naturally occurring flavonoid found in various fruits and vegetables, such as onions, strawberries, persimmons, apples and cucumbers. It has garnered attention for its potential protective effects against several health conditions, including osteoporosis^12^,^13^. Fisetin is recognized for its potent antioxidant properties, which aid in ROS and mitigating oxidative stress. Oxidative stress significantly contributes to bone loss and osteoporosis by damaging bone cells and impairing bone remodeling. By reducing oxidative stress, fisetin also helps protect against osteoblasts (bone-forming cells) and osteocytes (mature bone cells) from damage. This protective effect supports bone formation and maintenance, contributing to overall bone health.

Chronic inflammation is another factor that contributes to bone resorption and osteoporosis^12^,^14^. Fisetin has anti-inflammatory properties, which can help lower the levels of pro-inflammatory cytokines. By reducing inflammation, fisetin may help decrease the activity of osteoclasts (bone-resorbing cells), thereby preventing excessive bone loss and maintaining bone density.

Fisetin has been shown to modulate key signaling pathways involved in bone remodeling. Notably, it affects the RANK/RANKL/OPG pathway, which is essential for maintaining the balance between bone resorption and bone formation. It may inhibit the differentiation and activity of osteoclasts induced by receptor activator of nuclear factor-kappa B ligand (RANKL), while also promoting the expression of osteoprotegerin (OPG), a decoy receptor that binds to RANKL and prevents it from activating RANK^15^,^16^. This action helps maintain a healthy balance between bone resorption and formation, protecting against bone loss. Fisetin also appears to promote the activity and differentiation of osteoblasts, the cells responsible for bone formation. By enhancing osteoblast function, fisetin supports bone mineralization and the overall strength of the skeletal system. This effect is crucial for preventing osteoporosis, particularly in conditions in which bone formation is impaired. Fisetin has been found to inhibit osteoclastogenesis, the process by which osteoclasts are formed^16^. By suppressing the formation and activity of osteoclasts, fisetin helps reduce bone resorption, thus preserving bone mass and density. Fisetin may also work synergistically with other nutrients or treatments to enhance bone health. Its combination with other flavonoids, vitamins, or minerals could potentially amplify its protective effects against osteoporosis.

In this experimental study, we investigated the protective effects of fisetin against I/R injury in rat ovaries and explored the underlying mechanisms.

## Methods

### Experimental

Female Swiss albino Wistar rats, weighing 220 ± 20 g; female and aged 12–14 weeks old, were used in this experimental study. They were kept under standard laboratory conditions with controlled temperature and humidity, and a 12-hour light/dark cycle. The rats had access to adequate water and food. The entire animal protocol was approved by the departmental animal ethics committee, and the rats were acclimatized in the laboratory for seven days.

### Surgical procedures

All surgical procedures were conducted under standard laboratory conditions. The rats were weighed and anesthetized with an intramuscular injection of ketamine (50 mg/kg) and xylazine (10 mg/kg). Once anesthetized, the rats were placed in a dorsal recumbent position, and the surgical area was disinfected and draped with sterile coverings. To prevent hypothermia, the surgery was performed at a controlled temperature of 36 ± 1°C. A midline longitudinal incision of approximately 2 cm was made in the lower abdominal region to expose the ovaries. To induce the ischemia/reperfusion (I/R) model, vascular clamps were placed about 1 cm below the adnexal structures to simulate ovarian torsion. After 2 hours of induced torsion, the clamps were removed to initiate reperfusion, and the abdominal incision was closed using 4-0 nylon sutures^6^.

### Experimental design

After acclimatization, the rats were randomly divided into groups (n = 6), with each group containing six rats:

- Group I: Normal control;
- Group II: ovarian I/R (OI/R);
- Group III: OI/R+ fisetin (5 mg/kg);
- Group IV: OI/R + fisetin (10 mg/kg);
- Group V: OI/R + fisetin (15 mg/kg).

The dosage for the tested groups was determined based on findings from previous studies^17^–^19^. The body weight was estimated of all group rats at regular time intervals. The ovary weight was estimated at the end of the study. After completion of the experimental study, the rats were euthanized without pain or distress using enhancing the dose of anesthesia (using the intraperitoneal administration of pentobarbital), and the ovaries tissue were successfully isolated for the histopathological analyses. The ovarian tissues were stored at -40°C for further analysis.

Blood samples were drawn from the retro-orbital area under anesthesia. The samples were centrifuged at 10,000 rpm for 15 minutes to separate the serum, which was then stored at -20°C for subsequent biochemical analysis.

### Hematological parameters

The hematological parameters such as lymphocytes, red blood cells (RBC), hemoglobin, white blood cells (WBC) platelet, and lymphocytes were estimated using the MINDray BC 2800 Auto Hematology Analyzer.

### Biochemical parameters

The level of hormonal parameters like follicle-stimulating hormone (FSH), luteinizing hormone (LH), estradiol (E2) and anti-Mullerian hormone (AMH) were determined using the enzyme linked immunosorbent assay (ELISA) kits (Sunlong Biotech Co., Ltd, Zhejiang, China).

The level of oxidative stress parameters such as catalase (CAT), GSH, glutathione peroxidase (GPx), superoxide dismutase (SOD) and MDA; cytokines tumor necrosis factor-α (TNF-α), interleukin-1β (IL-1β), interleukin-6 (IL-6), interleukin-10 (IL-10), interleukin-18 (IL-18); inflammatory parameters like cyclooxynase-2 (COX-2), iNOS, prostaglandin E2 (PGE2), nuclear kappa B factor (NF-κB), C reactive protein (CRP); apoptosis parameters viz., caspase-3, Bcl-2 and Bax were estimated using the ELISA kits following the manufacture instruction (R&D Systems, Minneapolis, MN, United States of America).

HO-1 and Nrf_2_ were determined using the manufacture instruction.

### Histological and histopathologic evaluation

The rats were euthanized, and their ovarian tissues were extracted and fixed in 10% neutral buffered formalin for 48 hours. Each ovarian tissue sample was then dehydrated, embedded in paraffin, sectioned using a microtome, and stained with hematoxylin and eosin (H&E).

The histopathology of the all-group rats was observed under the light microscope. For the histopathologic evaluation, we examined at least five microscopical fields. The criteria for ovary injury were vascular congestion, cell degeneration (granulosa cells), inflammation (neutrophil infiltration), and hemorrhage. Each tissue was scored on a scale ranging from 0–3 (none = 0, mild = 1, moderate = 2, and severe = 3). The analysis on the ovary section was conducted in a blinded fashion.

### mRNA expression

Total RNA was extracted from frozen tissue using RNeasy mini kits and an automated DNA/RNA extraction machine (Qiacube, Qiagen, SP, Brazil), following the manufacturer’s instructions. The purity of the RNA was assessed with a spectrophotometer at 260 nm. Complementary DNA (cDNA) was synthesized from 10 µL of total RNA using the high-capacity cDNA reverse transcription kit (Qiacube, Qiagen, SP, Brazil). mRNA expression levels were quantified using quantitative real-time polymerase chain reaction (qPCR) with the StepOnePlus Systems (Applied Biosystems, United States of America). For normalization, mRNA levels from a housekeeping gene were quantified. The primer sequences are listed in Table 1. PCR conditions were as follows: 2 minutes at 50°C, 10 minutes at 95°C, and 40 cycles of 15 seconds at 95°C and 1 minute at 60°C. The data are means ± standard deviation of two independent treatment.

**Table 1:** List of gene and primer sequences.

S.	Gene	Primers (5’ - 3’)	
		Forward	Reverse
1	TNF-α	AGGCTGCCCCGACTACGT	GACTTTCTCCTGGTATGAGATAGCAAA
2	IL-1β	TTCAGGCAGGCAGTATCACTC	GAAGGTCCACGGGAAAGACAC
3	IL-4	CCATATCCACGGATGCGACA	AAGCACCTTGGAAGCCCTAC
4	IL-10	CTTACTGACTGGCATGAGGATCA	GCAGCTCTAGGAGCATGTGG
5	Bcl-2	CACTCGACCTTGTTTCTTCCAG	TCCTAACCCCTTGCTCTGCTT
6	Caspase-3	GGAGGCTGACTTCCTGTATGCTT	CCTGTTAACGCGAGTGAGAATG
7	Bax	GATGGCAACTTCAACTGGG	CCGAAGTAGGAGAGGAGGC
8	iNOS	TGTGACACACAGCGCTACAA	TGTTGAAGGCGTAGCTGAAC
9	eNOS	CTGCTGCCCGAGATATCTTC	CTGGTACTGCAGTCCCTCCT
10	MyD88	CGCATGGTGGTGGTTGTT	CGCTTCTGTTGGACACCT
11	TLR4	CTGTATTCCCTCAGCACTCTTGATT	CTGTATTCCCTCAGCACTCTTGAT
12	TRAF6	GGGAACGATACGCCTTACAA	CTCTGTCTTAGGGCGTCCAG
13	NF-κB	ACACTGGAAGCACGGATGAC	TGTCTGTGAGTTGCCGGTCT
14	IKKα	GTCAGGACCGTGTTCTCAAGG	GCTTCTTTGATGTTACTGAGGGC
15	Nrf2	GCCTTCCTCTGCTGCCATTAGT	TCGGCTGGGACTTGTGTTCAGT
16	HO-1	ACCCCACCAAGTTCAAACAGC	CCTCTGGCGAAGAAACTCTGTC
17	β-actin	GCTCCTCCTGAGCGCAAGTA	CAGCTCAGTAACAGTCCGCC

TNF-α: tumor necrosis factor-α; IL: interleukin; iNOS: inducible nitric oxide synthetase; eNOS: endothelial nitric oxide synthase; NF-κB: nuclear kappa B factor; IKKα: inhibitor of kappaB kinase alpha; Nrf2: nuclear factor erythroid 2-related factor 2; HO-1: heme oxygenase-1; MyD88: myeloid differentiation primary response 88, TLR4: Toll-like receptor 4; TRAF6: tumor necrosis factor receptor associated factor 6.

### Statistical analysis

One-way analysis of variance (ANOVA) was utilized for statistical analysis, followed by Dunnett’s multiple comparison test. Data from the study are presented as means ± standard error of the mean. GraphPad Prism software (version 8) was utilized for the analysis. Group differences were deemed significant if *p* < 0.05.

## Results

### Body weight and ovary weight

**Figure 1:**
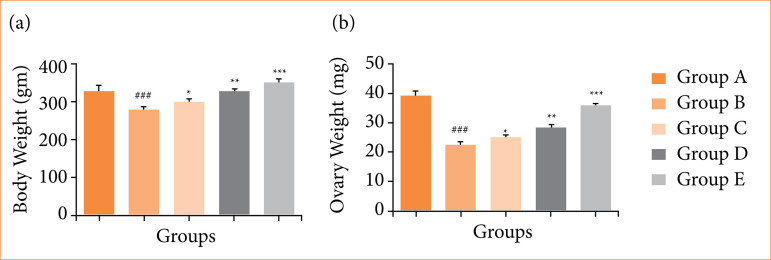
Effect of fisetin treatment on body weight and ovary weight in ovarian ischemia-reperfusion injury in rats. **(a)** Body weight; **(b)** ovary weight. Results are shown as means ± standard deviation.

Rats in the OI/R group experienced a decrease in body weight, whereas fisetin treatment led to a significant (*p* < 0.001) improvement in body weight (Fig. 1a). As shown in Fig. 1b, ovary tissue weight was reduced, but fisetin treatment significantly (*p* < 0.001) increased it.

###*p* < 0.001 compared with the normal control; *p < 0.05, **p < 0.01 and ***p < 0.001 compared with the ovarian ischemia-reperfusion injury group rats. Source: Elaborated by the authors.

### Hormones level

**Figure 2:**
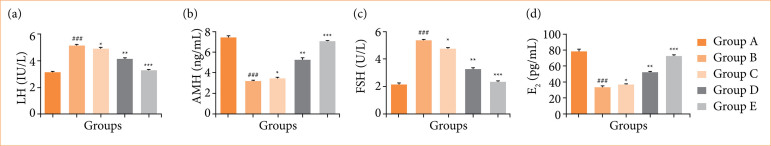
Effect of fisetin treatment on the hormone level in ovarian ischemia-reperfusion injury in rats. **(a)** Luteinizing hormone (LH), **(b)** anti-Mullerian hormone (AMH), **(c)** follicle-stimulating hormone (FSH), **(d)** estradiol (E2). Results are shown as means ± standard deviation.

Rats in the OI/R injury group exhibited altered hormone levels, including LH (Fig. 2a), AMH (Fig. 2b), FSH (Fig. 2c), and E2 (Fig. 2d). Fisetin treatment significantly (*p* < 0.001) restored these hormone levels.

###*p* < 0.001 compared with the normal control; *p < 0.05, **p < 0.01 and ***p < 0.001 compared with the ovarian ischemia-reperfusion injury group rats. Source: Elaborated by the authors.

### Oxidative stress parameters

**Figure 3:**
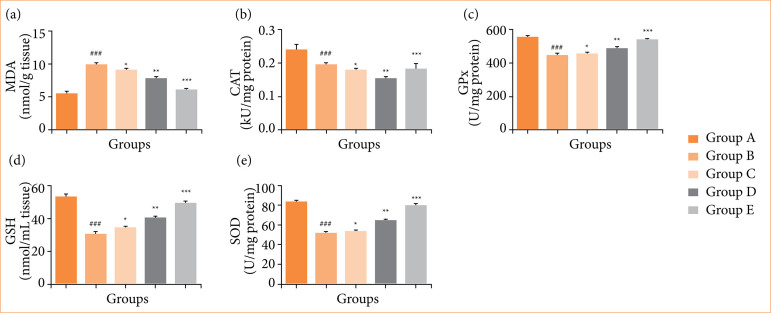
Effect of fisetin treatment on the antioxidant parameters in ovarian ischemia-reperfusion injury in rats. **(a)** malondialdehyde (MDA), **(b)** catalase (CAT), **(c)** glutathione peroxidase (GPx), **(d)** reduced glutathione (GSH), **(f)** superoxide dismutase (SOD). Results are shown as means ± standard deviation.

Comparing the OI/R group to the normal group revealed alterations in oxidative stress parameters, including MDA (Fig. 3a), CAT (Fig. 3b), GPx (Fig. 3c), GSH (Fig. 3d), and SOD (Fig. 3e). Fisetin treatment significantly (*p* < 0.001) restored the level of oxidative stress parameters.

###*p* < 0.001 compared with the normal control; *p < 0.05, **p < 0.01 and ***p < 0.001 compared with the ovarian ischemia-reperfusion injury group rats. Source: Elaborated by the authors.

### Hematological parameters

**Figure 4:**
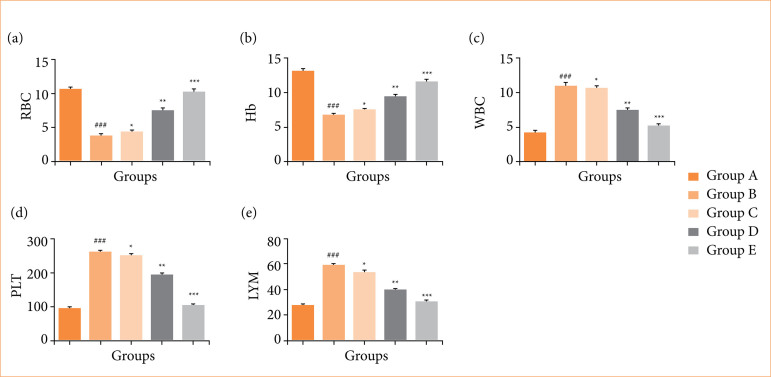
Effect of fisetin treatment on the level of hematological parameters in ovarian ischemia-reperfusion injury in rats. **(a)** red blood cell (RBC), **(b)** hemoglobin (Hb), **(c)** white blood cell (WBC), **(d)** platelet (PLT), **(e)** lymphocyte (LYM). Results are shown as means ± standard deviation.

The OI/R group exhibited changes in hematological levels, including RBC (Fig. 4a), hemoglobin (Fig. 4b), WBC (Fig. 4c), platelet (Fig. 4d), and lymphocytes (Fig. 4e). Fisetin treatment significantly (*p* < 0.001) restored these hematological parameters.

###*p* < 0.001 compared with the normal control; *p < 0.05, **p < 0.01 and ***p*p* < 0.001 compared with the ovarian ischemia-reperfusion injury group rats. Source: Elaborated by the authors.

### Cytokines and inflammatory parameters

**Figure 5:**
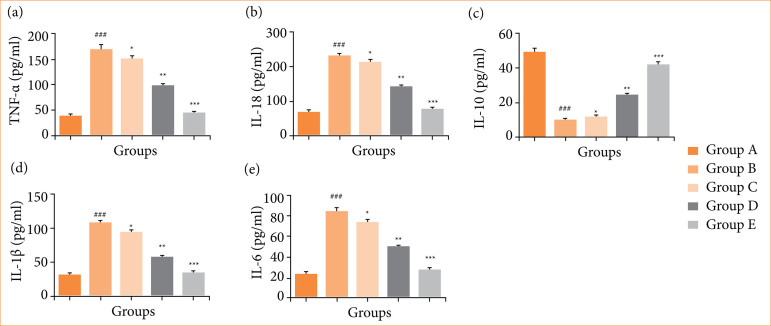
Effect of fisetin treatment on the level of inflammatory cytokines parameters in ovarian ischemia-reperfusion injury in rats. **(a)** tumor necrosis factor-α (TNF-α), **(b)** interleukin-18 (IL-18), **(c)** interleukin-10 (IL-10), **(d)** interleukin-1β (IL-1β), **(e)** interleukin-6 (IL-6). Results are shown as means ± standard deviation.

Rats in the OI/R group showed altered cytokine levels, including TNF-α (Fig. 5a), IL-18 (Fig. 5b), IL-10 (Fig. 5c), IL-1β (Fig. 5d), and IL-6 (Fig. 5e), indicating inflammation in the ovary tissue. Fisetin treatment significantly (*p* < 0.001) restored these cytokine levels.

###*p* < 0.001 compared with the normal control; *p < 0.05, **p < 0.01 and ***p < 0.001 compared with the ovarian ischemia-reperfusion injury group rats. Source: Elaborated by the authors.

**Figure 6:**
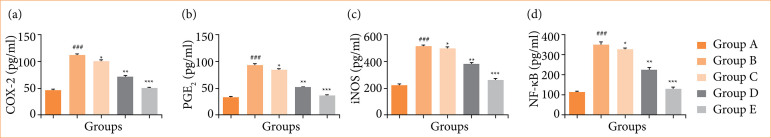
Effect of fisetin treatment on the level of inflammatory parameters in ovarian ischemia-reperfusion injury in rats. **(a)** cyclooxygenase-2 (COX-2), **(b)** prostaglandin E2 (PGE2), **(c)** inducible nitric oxide synthase (iNOS), **(d)** nuclear factor-κB (NF-κB). Results are shown as means ± standard deviation.

When comparing the OI/R group to the normal group, inflammatory parameters such as COX-2 (Fig. 6a), PGE2 (Fig. 6b), iNOS (Fig. 6c), and NF-κB (Fig. 6d) were elevated, but fisetin treatment significantly (*p* < 0.001) reduced these levels.

###*p* < 0.001 compared with the normal control; *p < 0.05, **p < 0.01 and ***p < 0.001 compared with the ovarian ischemia-reperfusion injury group rats. Source: Elaborated by the authors.

**Figure 7:**
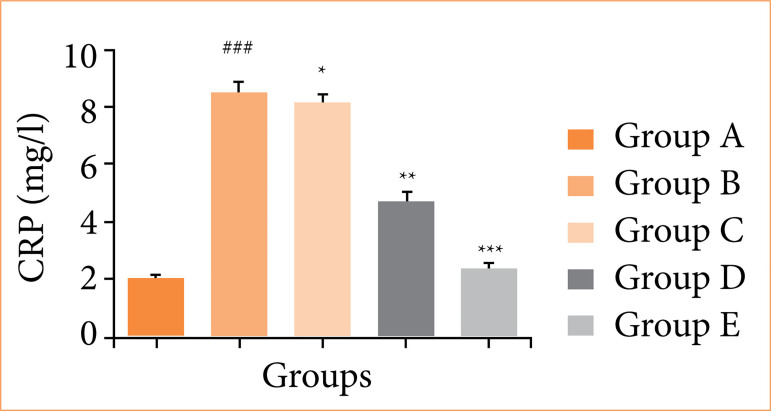
Effect of fisetin treatment on the level of C reactive protein (CRP) in ovarian ischemia-reperfusion injury in rats. Results are shown as means ± standard deviation.

Figure 7 shows that CRP levels were elevated in the OI/R group rats, and fisetin treatment significantly (*p* < 0.001) decreased these levels.

###*p* < 0.001 compared with the normal control; *p < 0.05, **p < 0.01 and ***p < 0.001 compared with the ovarian ischemia-reperfusion injury group rats. Source: Elaborated by the authors.

### Apoptosis parameters

**Figure 8:**
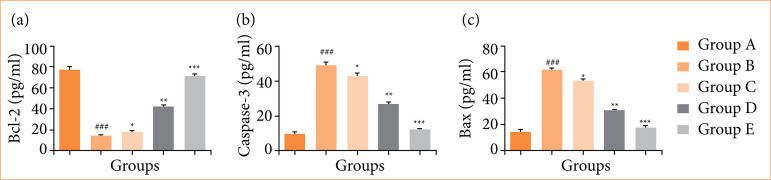
Effect of fisetin treatment on the apoptosis parameters in ovarian ischemia-reperfusion injury in rats. **(a)** Bcl-2, **(b)** caspase-3, **(c)** Bax. Results are shown as means ± standard deviation.

Rats in the OI/R group exhibited altered levels of apoptosis markers, including Bcl-2 (Fig. 8a), caspase-3 (Fig. 8b), and Bax (Fig. 8c). Fisetin treatment significantly (*p* < 0.001) restored these apoptosis parameters.

###*p* < 0.001 compared with the normal control; *p < 0.05, **p < 0.01 and ***p < 0.001 compared with the ovarian ischemia-reperfusion injury group rats. Source: Elaborated by the authors.

### Nrf2 and HO-1

**Figure 9:**
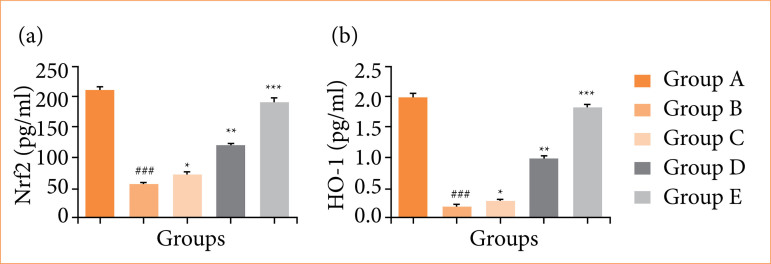
Effect of fisetin treatment on the level of Nuclear factor erythroid 2-related factor 2 (Nrf2) and heme oxygenase-1 (HO-1) in ovarian ischemia-reperfusion injury in rats. **(a)** Nrf2, **(b)** HO-1. Results are shown as means ± standard deviation.

Rats in the OI/R group exhibited reduced levels of Nrf2 (Fig. 9a) and HO-1 (Fig. 9b) compared to the normal group. Fisetin treatment led to a significant increase (*p* < 0.001) in the levels of both Nrf2 and HO-1.

###*p* < 0.001 compared with the normal control; *p < 0.05, **p < 0.01 and ***p < 0.001 compared with the ovarian ischemia-reperfusion injury group rats. Source: Elaborated by the authors.

### mRNA expression

**Figure 10:**
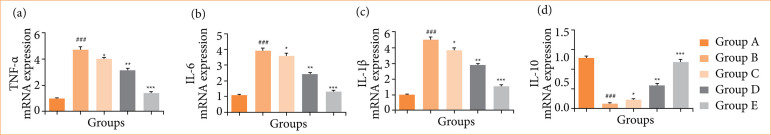
Effect of fisetin treatment on the mRNA expression of inflammatory cytokines in ovarian ischemia-reperfusion injury in rats. **(a)** tumor necrosis factor-α (TNF-α), **(b)** interleukin-6 (IL-6), **(c)** interleukin-1β (IL-1β), **(d)** interleukin-10 (IL-10). Results are shown as means ± standard deviation.

Rats in the OI/R group exhibited altered mRNA expression of TNF-α (Fig. 10a), IL-6 (Fig. 10b), IL-1β (Fig. 10c), IL-10 (Fig. 10d), Bax (Fig. 11a), Bcl-2 (Fig. 11b), caspase-3 (Fig. 11c), TLR4 (Fig. 12a), MyD88 (Fig. 12b), TRAF6 (Fig. 12c), eNOS (Fig. 13a), iNOS (Fig. 13b), NF-κB (Fig. 14a), iKKα (Fig. 14b), HO-1 (Fig. 15a), and Nrf2 (Fig. 15b), and fisetin treatment significantly (*p* < 0.001) modulated the mRNA expression.

###*p* < 0.001 compared with the normal control; *p < 0.05, **p < 0.01 and ***p < 0.001 compared with the ovarian ischemia-reperfusion injury group rats. Source: Elaborated by the authors.

**Figure 11:**
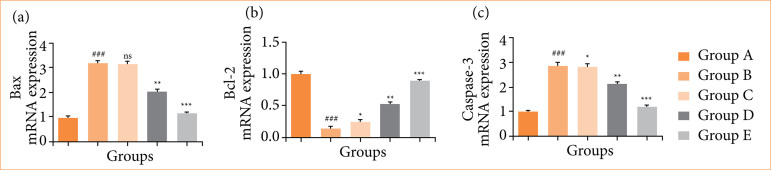
Effect of fisetin treatment on the mRNA expression of apoptosis in ovarian ischemia-reperfusion injury in rats. **(a)** Bax, **(b)** Bcl-2, **(c)** caspase-3. Results are shown as means ± standard deviation.

###*p* < 0.001 compared with the normal control; *p < 0.05, **p < 0.01 and ***p < 0.001 compared with the ovarian ischemia-reperfusion injury group rats. Source: Elaborated by the authors.

**Figure 12:**
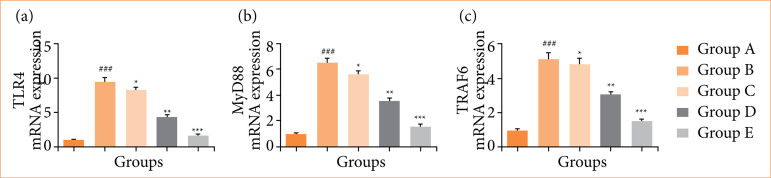
Effect of fisetin treatment on mRNA expression in ovarian ischemia-reperfusion injury in rats. **(a)** TLR4, **(b)** MyD88, **(c)** FTRAF6. Results are shown as means ± standard deviation.

###*p* < 0.001 compared with the normal control; *p < 0.05, **p < 0.01 and ***p < 0.001 compared with the ovarian ischemia-reperfusion injury group rats. Source: Elaborated by the authors.

**Figure 13:**
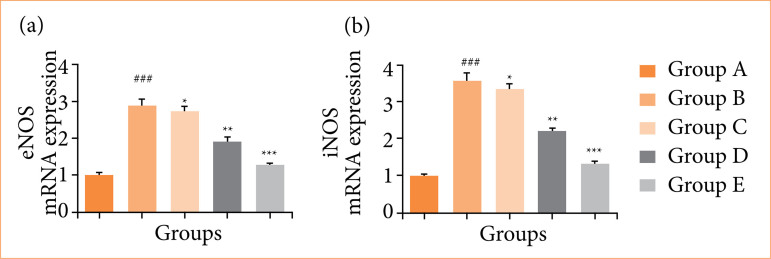
Effect of fisetin treatment on mRNA expression in ovarian ischemia-reperfusion injury in rats. **(a)** endothelial nitric oxide synthase (eNOS), **(b)** inducible nitric oxide synthase (iNOS). Results are showm as means ± standard deviation.

###*p* < 0.001 compared with the normal control; *p < 0.05, **p < 0.01 and ***p < 0.001 compared with the ovarian ischemia-reperfusion injury group rats. Source: Elaborated by the authors.

**Figure 14:**
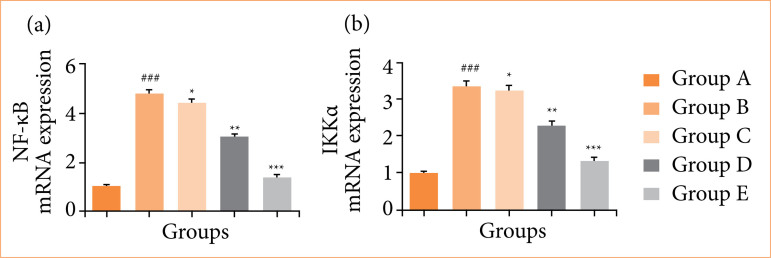
Effect of fisetin treatment on mRNA expression in ovarian ischemia-reperfusion injury in rats. **(a)** nuclear factor-kappa B (NF-κB), **(b)** inhibitory-κB kinase alpha (IKKα). Results are shown as means ± standard deviation.

###*p* < 0.001 compared with the normal control; *p < 0.05, **p < 0.01 and ***p < 0.001 compared with the ovarian ischemia-reperfusion injury group rats. Source: Elaborated by the authors.

**Figure 15:**
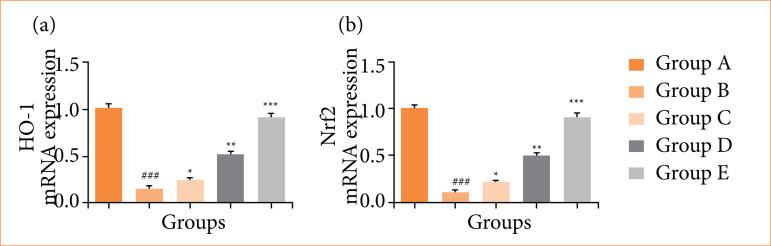
Effect of fisetin treatment on mRNA expression in ovarian ischemia-reperfusion injury in rats. **(a)** heme oxygenase-1 (HO-1), **(b)** nuclear factor erythroid 2-related factor 2 (Nrf2). Results are shown as means ± standard deviation.

###*p* < 0.001 compared with the normal control; *p < 0.05, **p < 0.01 and ***p < 0.001 compared with the ovarian ischemia-reperfusion injury group rats. Source: Elaborated by the authors.

### Histopathologic scores

**Figure 16:**
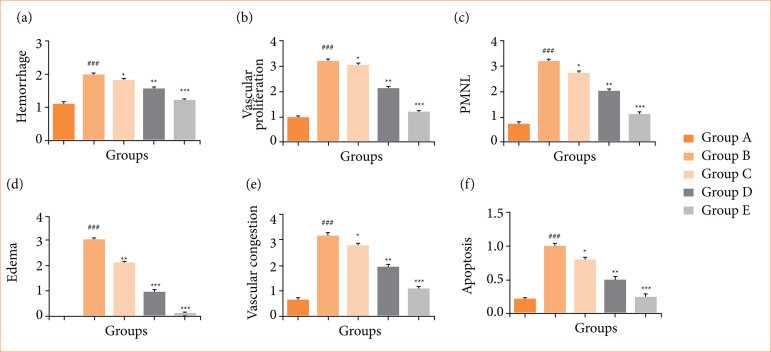
Effect of fisetin treatment on histopathologic scores in ovarian ischemia-reperfusion injury in rats. **(a)** hemorrhage, **(b)** vascular proliferation, **(c)** polymorphonuclear leukocytes (PMNL), (d) edema, (e) vascular congestion, (f) apoptosis. Results are shown as means ± standard deviation.

OI/R group rats exhibited the boosted histopathologic scores such as hemorrhage (Fig. 16a), vascular proliferation (Fig. 16b), PMNL (Fig. 16c), edema (Fig. 16d), vascular congestion (Fig. 16e), and apoptosis (Fig. 16f), and fisetin treatment significantly (*p* < 0.001) suppressed the histopathologic score.

###*p* < 0.001 compared with the normal control; *p < 0.05, **p < 0.01 and ***p < 0.001 compared with the ovarian ischemia-reperfusion injury group rats. Source: Elaborated by the authors.

## Discussion

Ovarian ischemia, a significant gynecological condition, arises when the ovaries twist, often due to factors such as ovarian masses or surgical interventions. Detorsion, which restores blood flow to the affected ovaries, can cause reperfusion injury^20^,^21^. This injury results from the sudden return of oxygenated blood, leading to oxidative stress, inflammation, and cellular damage. Therefore, conservative treatment before surgery is crucial to manage I/R injury^21^,^22^. Without antioxidant and anti-inflammatory treatment, ischemic ovarian damage can be more severe, highlighting the importance of conservative therapy.

AMH, FSH, and E_2_ are hormones integral to ovarian function, with their levels and interactions extensively studied in the context of OI/R Injury. AMH, produced by granulosa cells in ovarian follicles, serves as a marker of ovarian reserve, indicating the remaining number of oocytes^6^. It serves as a crucial marker for predicting reproductive lifespan and evaluating long-term ovarian function. In women with advanced OI/R injury, AMH levels are generally lower than in healthy individuals, as the disease can diminish ovarian reserve. Some studies propose that AMH could serve as an early detection marker for OI/R injury, particularly in premenopausal women, although its diagnostic utility remains under investigation.

FSH is essential for the growth and maturation of ovarian follicles, stimulating estrogen production and regulating the menstrual cycle^22^,^23^. Elevated FSH levels have been linked to the development of certain OI/R, especially in postmenopausal women. High FSH levels can enhance the proliferation of OI/R cells by increasing estrogen production, potentially contributing to tumor growth. E_2_, the primary form of estrogen produced by the ovaries, regulates the menstrual cycle, supports reproductive health, and is involved in the development of secondary sexual characteristics. Estradiol is implicated in the development of OI/R, as it can stimulate the growth of hormone-responsive ovarian tumors. Elevated estradiol levels are often found in women with OI/R, especially in estrogen receptor-positive tumors, indicating that estradiol may function as a growth factor for these cancers^6^,^21^,^22^,^24^. Rats in the OI/R group showed reduced levels of AMH and E2, along with elevated levels of FSH. Fisetin treatment significantly restored these hormone levels.

OI/R injury happens when blood flow to the ovaries is temporarily halted (ischemia) and then resumed (reperfusion). This process can cause significant tissue damage and inflammation, with several cytokines playing key roles in mediating these effects^25^. Cytokines are well-known low molecular weight proteins that facilitate communication between cells. They are crucial in balancing cell differentiation and proliferation, activating immune cells, ensuring cell survival, facilitating cell migration, and ultimately inducing apoptosis^26^. TNF-α is a pro-inflammatory cytokine rapidly produced in response to tissue injury. In OI/R injury, TNF-α contributes to the inflammatory response by attracting neutrophils and other immune cells to the injury site. It also stimulates the production of additional inflammatory cytokines and can induce apoptosis (programmed cell death) in ovarian cells.

Similarly, IL-1β is a potent pro-inflammatory cytokine involved in the early stages of the inflammatory response during I/R injury^21^,^24^. It activates endothelial cells, increases vascular permeability, and enhances the expression of adhesion molecules that facilitate the migration of immune cells to the injury site. IL-1β also promotes the production of other inflammatory mediators, contributing to the overall inflammatory cascade. IL-6 exhibits both pro-inflammatory and anti-inflammatory properties, making its role in OI/R injury complex. Initially, IL-6 acts as a pro-inflammatory mediator, triggering the acute-phase response and further recruitment of immune cells. However, IL-6 can also stimulate the production of anti-inflammatory cytokines and acute-phase proteins that help limit tissue damage. In OI/R injury, IL-6 regulates the balance between inflammation and tissue repair^27^. IL-10 is mainly an anti-inflammatory cytokine that plays a protective role in OI/R injury by inhibiting the production of pro-inflammatory cytokines such as TNF-α, IL-1, and IL-6. It helps limit inflammation and tissue damage by downregulating the immune response, promoting tissue repair, and reducing oxidative stress. Conversely, IL-18, a pro-inflammatory cytokine from the IL-1 family, contributes to the inflammatory response in OI/R injury by inducing the production of other cytokines like IFN-γ and enhancing the cytotoxic activity of immune cells. IL-18 can exacerbate tissue damage by promoting inflammation and oxidative stress. During the OI/R process, the balance between pro-inflammatory and anti-inflammatory cytokines determines the extent of tissue damage and the subsequent healing process^28^,^29^. The levels of cytokines are altered during OI/R injury, and fisetin has been shown to remarkably restore the levels of these inflammatory cytokines.

During OI/R injury, multiple molecular pathways contribute to tissue damage and inflammation. eNOS is an enzyme that produces NO in blood vessels. NO acts as a vasodilator, helping to regulate blood flow and maintain vascular homeostasis^30^. In OI/R injury, eNOS plays a protective role by promoting NO production, which improves blood flow during reperfusion, reduces oxidative stress, and limits tissue damage. Enhanced eNOS activity can help attenuate the inflammatory response and promote tissue recovery. In contrast, iNOS is induced under pathological conditions, including inflammation and oxidative stress^31^. In OI/R injury, iNOS produces large amounts of NO, which, in excess, can react with superoxide to form peroxynitrite, a potent oxidant that contributes to tissue damage. iNOS-derived NO is associated with increased inflammation, oxidative stress, and cell death, exacerbating ovarian I/R injury. TLR4 is a pattern recognition receptor crucial for the innate immune response, as it identifies pathogen-associated molecular patterns (PAMPs) and damage-associated molecular patterns (DAMPs). In OI/R injury, TLR4 is activated by DAMPs from damaged ovarian tissue, triggering signaling pathways that generate pro-inflammatory cytokines. This intensifies the inflammatory response and contributes to tissue damage. MyD88, an adaptor protein, is vital for signaling in most TLRs, including TLR4. When TLR4 is activated in OI/R injury, MyD88 transmits signals that activate NF-κB and other transcription factors, leading to the production of pro-inflammatory cytokines and chemokines, which further promote inflammation and exacerbate tissue damage^32^. TRAF6 is an adaptor protein that plays a role in the signaling pathways of TLRs and other receptors, including IL-1 receptors. In OI/R injury, TRAF6 is a crucial mediator of the MyD88-dependent pathway, aiding in the activation of NF-κB and mitogen-activated protein kinases (MAPKs), which leads to the transcription of pro-inflammatory genes^33^. TRAF6 is an adaptor protein involved in the signaling pathways of TLRs and other receptors, including IL-1 receptors. In OI/R injury, TRAF6 is crucial in the MyD88-dependent pathway, aiding the activation of NF-κB and MAPKs, which subsequently leads to the transcription of pro-inflammatory genes^32^. Treatment with fisetin has been shown to significantly downregulate the mRNA expression of these molecules, suggesting its protective effect against ovarian injury.

HO-1 and Nrf2 are crucial components of the cellular defense mechanism against oxidative stress and inflammation. HO-1 is an inducible enzyme that offers protection by degrading heme into biliverdin, carbon monoxide (CO), and free iron in response to oxidative stress and inflammation^34^,^35^. HO-1 reduces oxidative damage, inflammation, and apoptosis in ovarian tissue during I/R injury. By breaking down pro-oxidant heme and generating protective molecules, HO-1 plays a crucial role in minimizing tissue damage and promoting recovery. Nrf2 is a transcription factor that regulates the expression of various antioxidant and cytoprotective genes. Under normal conditions, Nrf2 is sequestered in the cytoplasm by its inhibitor, Kelch-like ECH-associated protein 1 (Keap1)^34^. Under oxidative stress, Nrf2 dissociates from Keap1, translocates to the nucleus, and binds to antioxidant response elements (AREs) in the promoter regions of target genes, such as HO-1^35^. Nrf2 activation is essential for the cellular response to oxidative stress and inflammation. By enhancing the expression of antioxidant enzymes like HO-1 and glutathione peroxidase, Nrf2 helps protect ovarian tissue from oxidative damage induced by I/R injury^36^. The Nrf2 pathway is essential for reducing inflammation, preventing apoptosis, and promoting tissue repair in the ovaries^37^. In OI/R injury, both HO-1 and Nrf2 are vital protective mechanisms against oxidative stress and inflammation. By upregulating HO-1 and other antioxidant genes, Nrf2 mitigates the damage caused by I/R injury, thereby enhancing tissue survival and recovery^37^,^38^. During OI/R injury, the mRNA expression of HO-1 and Nrf2 was suppressed, but fisetin significantly boosted their mRNA expression.

## Conclusion

Dose-dependent fisetin treatment significantly improved body and ovary weights, along with favorable alterations in hematological parameters (RBC, WBC, hemoglobin, platelet, lymphocytes), antioxidant levels (MDA, SOD, GSH, GPx, CAT), and cytokine profiles. Additionally, fisetin effectively modulated inflammatory markers (COX-2, iNOS, PGE2, NF-κB, CRP) and apoptosis-related parameters (Bcl-2, Bax, caspase-3). The treatment also led to significant changes in the mRNA expression of key genes associated with inflammation, apoptosis, and oxidative stress. Moreover, fisetin considerably reduced pathological features such as hemorrhage, vascular proliferation, PMNL infiltration, edema, vascular congestion, and apoptosis, highlighting its potential therapeutic value in improving ovarian health.

## Data Availability

The data will be available on the request to the corresponding author.
